# The Thermoelectric Properties of *n*-Type Bismuth Telluride: Bismuth Selenide Alloys Bi_2_Te_3−*x*_Se_*x*_

**DOI:** 10.34133/2020/4361703

**Published:** 2020-03-31

**Authors:** Ian T. Witting, Francesco Ricci, Thomas C. Chasapis, Geoffroy Hautier, G. Jeffrey Snyder

**Affiliations:** ^1^Department of Materials Science and Engineering, Northwestern University, 2220 Campus Drive, Cook Hall 2036, Evanston, IL 60208, USA; ^2^Institute of Condensed Matter and Nanosciences, Université Catholique de Louvain, Louvain-la-Neuve 1348, Belgium

## Abstract

Alloying bismuth telluride with antimony telluride and bismuth selenide for *p*- and *n*-type materials, respectively, improves the thermoelectric quality factor for use in room temperature modules. As the electronic and thermal transports can vary substantially, the alloy composition is a key engineering parameter. The *n*-type Bi_2_Te_3-__*x*_Se_*x*_ alloy lags its *p*-type counterpart in thermoelectric performance and does not lend itself as readily to simple transport modeling which complicates engineering. Combining literature data with recent results across the entire alloy composition range, the complex electronic structure dynamics and trends in lattice thermal conductivity are explored. Spin-orbit interaction plays a critical role in determining the position and degeneracy of the various conduction band minima. This behavior is incorporated into a two-band effective mass model to estimate the transport parameters in each band. An alloy scattering model is utilized to demonstrate how phonon scattering behaves differently on either side of the intermediate ordered compound Bi_2_Te_2_Se due to chalcogen site occupancy preference. The parametrization of the electronic and thermal transports presented can be used in future optimization efforts.

## 1. Introduction

Bismuth telluride is the dominant thermoelectric material for applications near room temperature due to its inherently low lattice thermal conductivity and high electronic weighted mobility [1]. Its performance is ultimately limited by the deleterious effects of thermally generated minority carriers as a result of its small band gap (0.14 eV) [2]. This effect can be partially mitigated by doping more heavily than what would be ideal considering the transport of majority carriers alone. Alloying with Sb_2_Te_3_ for *p*-type or Bi_2_Se_3_ for *n*-type materials can also reduce bipolar effects by increasing the band gap, but the impact on thermal and electric transports must also be considered [3–6]. Thermal conductivity can be significantly reduced by the enhanced scattering of high frequency phonons by introducing mass contrast and bonding changes [7–9]. Charge carrier mobility can also be disrupted during alloying while changes in electronic structure could prove beneficial or detrimental [10, 11]. Engineering bismuth telluride-based thermoelectrics require simultaneous consideration of these details.

The *p*-type Bi_2_Te_3_-Sb_2_Te_3_ alloy has been well characterized and modeled. The thermal conductivity variation with composition is well described using a mass contrast alloy scattering model where composition varies on a single cation site [12]. The valence band structure for both binary compounds is similar with the primary difference being a switch in the order of the two valence band maxima [1]. These band edges cross in energy near Bi_0.5_Sb_1.5_Te_3_ resulting in an enhancement of the thermoelectric power factor [13–15]. This convergence in energy combined with a significant reduction in thermal conductivity leads to this being the optimum composition.

The *n*-type Bi_2_Te_3_-Bi_2_Se_3_ alloy is less straightforward. Mass contrast alloy scattering modeling does not match thermal conductivity values reported in the literature, and there is wide variability in qualitative trends reported [8, 9, 12, 16]. The electronic band structure of Bi_2_Te_3_ is comprised of conduction and valence band extrema with high valley degeneracy while the structure of Bi_2_Se_3_ is far simpler with a direct gap between singly degenerate extrema at the Γ point [1, 17]. This dramatic difference in Fermi surface complexity along with a peak in the optical band gap near the intermediate composition Bi_2_Te_2_Se suggests that complex band dynamics are in play [4, 5]. There has recently been a great deal of interest in the electronic structure of Bi_2_Te_3−*x*_Se_*x*_ alloys as these materials are also topological insulators [18–23]. Accordingly, these studies have primarily focused upon the surface states and the variation with composition of the energy level of the Dirac point relative to band edges. While the bulk structures of the endmembers Bi_2_Te_3_ and Bi_2_Se_3_ have been extensively studied, a comprehensive description of the dynamics in the alloy has not been reported.

In this report, we aim to explain long standing unresolved issues in the Bi_2_Te_3−*x*_Se_*x*_ alloy system. Combining experimental results from the literature and new electronic structure calculations, we describe how the electronic structure changes with composition. Electronic structure calculations for this system are highly sensitive to the magnitude of the spin-orbit interaction which has complicated resolving these issues. This is then used to parameterize the electronic transport in *n-*type Bi_2_Te_3−*x*_Se_*x*_ in an effective mass model. In addition, we present an alloy scattering model of the lattice thermal conductivity which provides clarity regarding compositional trends by accounting for the occupancy preference of two inequivalent chalcogen sites.

## 2. Crystal Structure

**Figure 1:**
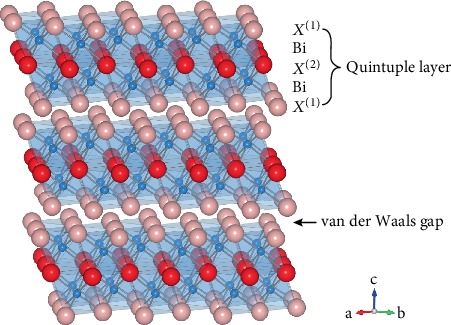
The crystal structure of Bi_2_Te_3−*x*_Se_*x*_ is comprised of quintuple layers of *X*^(1)^ − Bi − *X*^(2)^ − Bi − *X*^(1)^ where *X* represents either Te or Se and the number in parentheses designates between the two inequivalent sites. The *X*^(2)^ site is preferentially occupied by Se, and the alloy forms an ordered compound at the Bi_2_Te_2_Se composition.

Bismuth telluride, bismuth selenide, and all intermediate alloys Bi_2_Te_3−*x*_Se_*x*_ have the tetradymite crystal structure in the symmetry group $\text{R}\bar{3}\text{m}$ (Figure 1) [24, 25]. The material is comprised of repeating quintuple layers of *X*^(1)^–Bi–*X*^(2)^–Bi–*X*^(1)^ where the number in parentheses designates two inequivalent chalcogen (*X*) sites. Bismuth atoms are octahedrally coordinated by chalcogen atoms, and the *X*^(2)^ site atoms are octahedrally coordinated by Bi. The *X*^(1)^ site atoms are covalently bonded with three Bi atoms and by weaker van der Waals bonds with three other *X*^(1)^ atoms. The Bi-*X*^(1)^ bond length is close to the expected covalent bond length, while the Bi-*X*^(2)^ bond is longer and near the value expected for ionic bonding [25, 26]. The *X*^(2)^ site is preferentially occupied by Se [25–28]. This site selectivity has been attributed to a reduction in bond angle strain and to the more electronegative Se occupying the site with the highest cation coordination [28, 29]. This is a key difference from the *p*-type Bi_2_Te_3_-Sb_2_Te_3_ alloy system where Bi and Sb occupy equivalent sites without preference.

The difference in bonding at each chalcogen site and the occupancy preference has significant consequences for the electrical and thermal properties of the system. The preference results in the formation of an ordered compound at Bi_2_Te_2_Se which acts as a demarcation point between which the chalcogen site is being changed during alloying. There is some disagreement in the literature as to whether the ordered compound is stable or if solid state phase separation occurs as observed in Bi_2_Te_2_S [29]. In Bi_2_Te_2_S, this separation occurs to relieve bond angle strains that would otherwise occur in the ordered Te^(1)^–Bi–S^(2)^–Bi–Te^(1)^ compound. Ordering of the Bi_2_Te_2_Se phase has been observed in numerous studies [25, 26, 30, 31], but others report that the phase will separate into two compounds with compositions near *x* = 0.5 and *x* = 1.4 if annealed at temperatures near 300°C for extended periods [32, 33]. If phase separation does occur, it may be kinetically inhibited at temperatures typically used for thermoelectric or topological insulator applications. This report assumes that this inhibition occurs in typical thermoelectric samples.

The layered structure of the tetradymites results in significant anisotropy in transport properties. In the *n*-type Bi_2_Te_3−*x*_Se_*x*_ alloy system, the ratio of the electrical conductivity within the *ab* planes to that in the direction parallel to the *c*-axis ranges from 3 to 7, while the thermal conductivity anisotropy ratio is closer to 2 [34–36]. As the Seebeck coefficient is nearly isotropic when not demonstrating intrinsic conduction, the *zT* ratio is 2-3 necessitating the use of single or oriented polycrystals to maximize performance. This contrasts with the *p-*type Bi_2_Te_3_-Sb_2_Te_3_ alloy system where the electrical and thermal conductivity ratios are both near 2-3 resulting in a nearly isotropic *zT* [37]. This isotropy in the *p*-type system allows for significant improvements in *zT* to be made by nanostructuring for the reduction of lattice thermal conductivity. [38, 39] Nanostructuring can produce benefits in the *n*-type alloys as well, but they are smaller and require additional processing steps to preferentially orient the grains [40–44]. Figure-of-merit values with nanotexturing have been reported between *zT* = 1.4‐1.8 in *p*-type Bi_0.5_Sb_1.5_Te_3_, but only in the range of 1-1.2 for Bi_2_Te_3−*x*_Se_*x*_ alloys with *x* < 1. The experimental data presented in this study come from single or highly oriented large grain polycrystals, and properties are measured along the high *zT* direction in the *ab* planes.

## 3. Electronic Transport

**Figure 2:**
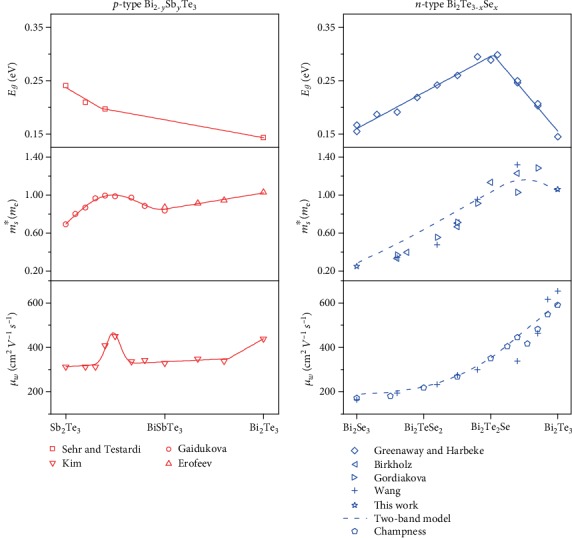
The band gap and electronic transport in *p*- and *n*-type bismuth telluride alloys show important differences in compositional trends due to the details of their complex band dynamics. In Bi_2−*y*_Sb_*y*_Te_3_, a change in slope of the band gap with composition coincides with a peak in Seebeck mass and weighted mobility when modeled using a single band [6, 13–15]. This correlation is explained by a crossing of two valence bands in energy with composition [13]. In Bi_2_Te_3−*x*_Se_*x*_, a peak in band gap does not coincide in composition with the peak in effective mass and no peak in weighted mobility is observed [4, 8, 16, 53, 54]. This suggests that the underlying causes differ for the mass and band gap peaks. The solid lines in this figure are simple guides to the eye, while the dashed lines represent single band evaluation of the simplified two-conduction band effective mass model discussed within the text and evaluated for a constant doping level of 5∗10^19^ cm^−3^.

The electronic transport properties vary significantly with alloy composition as shown in Figure 2. Pisarenko plot fits of Seebeck versus Hall carrier concentration using a single valley effective mass model assuming deformation potential scattering find that the Seebeck effective mass, *m*_*S*_^∗^, decreases from 1.06 *m*_e_ for Bi_2_Te_3_ to 0.25 *m*_e_ for Bi_2_Se_3_ [1, 8, 35, 45–50]. Similarly, the weighted mobility, *μ_w_*, which sets the maximum achievable power factor, decreases monotonically from 590 to 170 cm^2^V^−1^ s^−1^ going from Bi_2_Te_3_ to Bi_2_Se_3_ [16]. This is likely due to a loss of Fermi surface complexity by a decrease in valley degeneracy and/or by a decrease of the conduction anisotropy of each valley [51].

In the Bi_2_Te_3_-Sb_2_Te_3_ alloy system, a peak in the effective mass and an abrupt change in the band gap slope with composition is observed near Bi_0.5_Sb_1.5_Te_3_ [6, 52]. This has been attributed to the crossing in energy with composition of two *N*_*v*_ = 6 valence bands [13]. In the *n*-type alloy system, there is a peak in the band gap with alloy composition very near Bi_2_Te_2_Se and also a peak in Seebeck effective mass of ~1.30 *m*_e_ near Bi_2_Te_2.5_Se_0.5_ (Figure 2) [1, 4, 5, 8, 53–55]. As the compositions for two peaks do not coincide, the transport behavior cannot be described by a simple crossing of two conduction bands and a more detailed investigation is required.

## 4. Electronic Structure

The electronic structure of the Bi_2_Te_3−*x*_Se_*x*_ alloy system has been studied extensively due to the importance of these materials as thermoelectrics and topological insulators; however, the complete picture of dynamics of key band edges has not been laid out. This section summarizes the experimental and theoretical work for these alloys which is then used to inform our picture of how the band structure evolves with composition. It should be noted that both experimental and theoretical characterizations of the band structure are performed at low temperatures, and positions and shapes of band extrema may be shifted at temperatures where these materials operate as thermoelectrics [4, 56].

**Figure 3:**
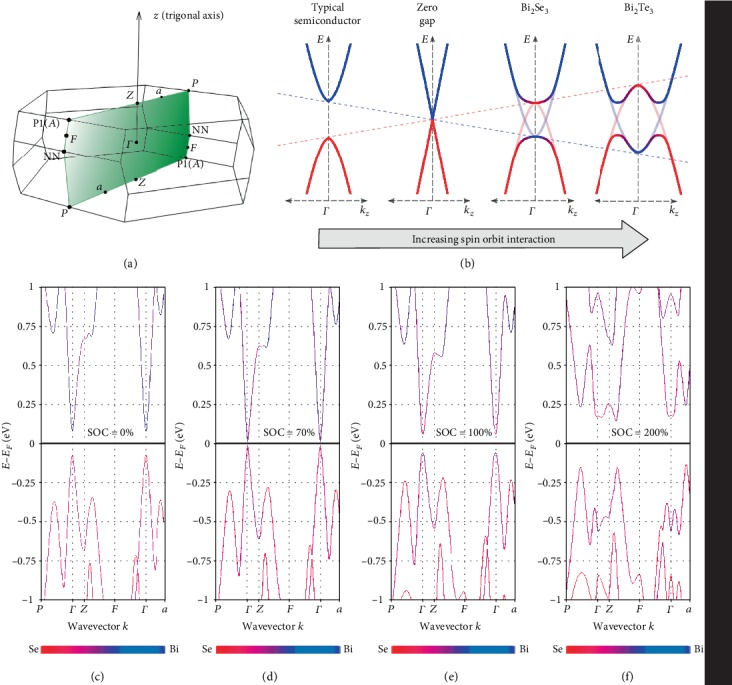
Schematic illustration of the impact of the spin-orbit interaction induced band overlap in the Bi_2_Te_3−*x*_Se_*x*_ alloy system. The band extrema relevant for transport all exist within the bisectrix plane of the Brillouin zone (a). Two important locations within this plane are not indicated due to their position shifting slightly with alloy composition. The *f* point lies slightly offset from the *ZF* line, while the *z* lies along Γ*Z*. A simplified view of the effect of increasing the spin-orbit interaction is shown in (b). The gap of a typical semiconductor between cation conduction band states and anion valence band states closes with increasing spin-orbit interaction until the bands overlap. Due to the opposite parity of these specific bands in Bi_2_Se_3_ and Bi_2_Te_3_, an anticrossing occurs at the overlap creating a band gap and change of character for states at Γ. In Bi_2_Te_3_, the inversion is significant enough to invert the curvature at Γ and shifts the CBM towards a higher degeneracy point. Away from Γ, the energy level of other extrema shifts as shown by electronic structure calculations in (c–f) for Bi_2_Se_3_ with varying strengths of spin-orbit interaction. Doubling the strength of the interaction causes the CB extremum at Γ to shift to *z* and slightly above a sixfold valley at f, strongly resembling the calculated structure for Bi_2_Te_3_ (Figure 4).

Experimental characterization of the Bi_2_Te_3_ conduction band Fermi surface has found the conduction band minimum to be sixfold degenerate and nonparabolic [57–64]. Calculations have found this minimum to be within the bisectrix plane (Figure 3(a), highlighted) and slightly displaced from the *ZF* line, a point we designate as *f* [1]. Shubnikov-de Haas (SdH) measurements also detected a second, heavier band edge only 25 meV above the CBM or at a carrier concentration of *n*_*H*_ = −1/*eR*_*H*∞_ = 2∗10^18^ cm^−3^ [64]. This pocket has been calculated to be twofold degenerate and lying on the Γ*Z* line, a point designated here as *z*. When both the *f* and *z* pockets are occupied, the conduction band Fermi surface forms a “double tripod” structure which has been observed by angle-resolved photoemission spectroscopy (ARPES) [20]. A third pocket higher in energy than *z* is predicted to be present in a position slightly displaced from the Γ*a* line making it sixfold degenerate. Experimental characterization of this edge has not been made; however, it cannot be ruled out that at operating temperatures and elevated doping, it potentially plays a role in transport.

**Figure 4:**
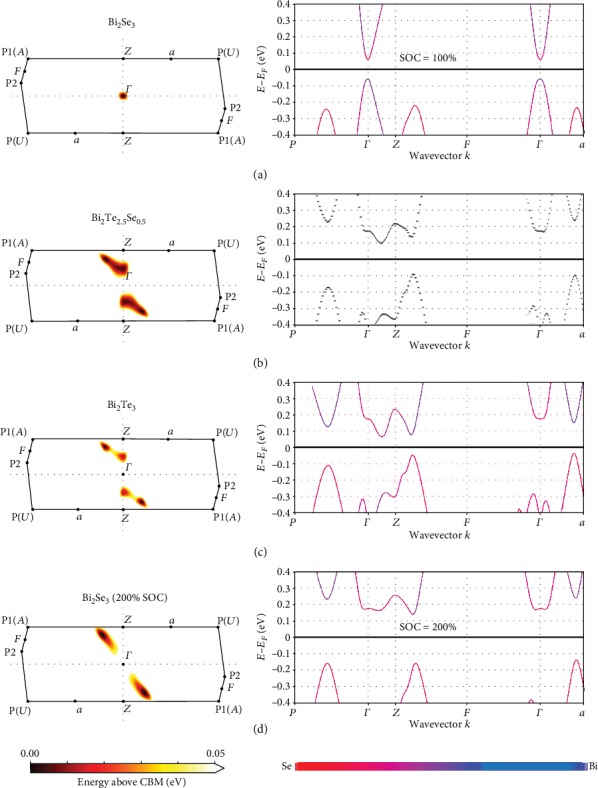
Fermi surface maps in the bisectrix plane illustrate the variation in positions and energies of the conduction band extrema with alloy composition. In Bi_2_Se_3_ (a), the CBM is singly degenerate at Γ, while in Bi_2_Te_3_ (c), the CBM is at the sixfold f point slightly displaced from the *ZF* line with a second twofold pocket at z along the Γ*Z* line. In alloys near Bi_2_Te_2.5_Se_0.5_ (b), the pockets at f and z are at almost the same energy level. Varying the alloy composition manipulates the magnitude of the spin-orbit interaction and shifts the energy and positions of various extrema. This is illustrated in (d) where doubling the spin-orbit interaction in Bi_2_Se_3_ calculations results in a CBM at f as in Bi_2_Te_3_.

ARPES and SdH measurements have found the CBM in Bi_2_Se_3_ to be a single ellipsoidal valley centered at Γ and elongated along the trigonal axis [22, 65, 66]. At a Fermi level 160 meV above the CBM or *n*_*H*_ = 10^19^ cm^−3^, a steep increase in *n*_*H*_ with the Fermi level is observed which may indicate the filling of a second band edge having a larger density-of-states [65, 67]. Electronic structure calculations for Bi_2_Se_3_ (Figures 3(c) and 4(a)) qualitatively match with experimental observations. Near the CBM edge, the Fermi surface is a simple ellipsoid centered at Γ. Our calculations find a second band edge along the *ZF* line (Figure 3(e)), similar to the CBM of Bi_2_Te_3_ at *f* which may explain the rise in density-of-states observed by SdH 160 meV above the CBM. To the best of the authors' knowledge, this second edge has not been directly imaged by ARPES. Chen et al. used ARPES to observe the conduction band up to a Fermi level of 150 meV above the minimum at Γ [22]. This is below where the second edge was potentially detected by SdH.

Köhler et al. performed Shubnikov-de Haas measurements on Bi_2_Te_3−*x*_Se_*x*_ alloy samples near the binary endpoints: *x* < 0.3 and *x* > 2.4 [68]. On the Bi_2_Te_3_ side, the energy separation between the CBM (*f*) and the second band edge (*z*) linearly decreases to zero at *x* = 0.285 ± 0.015. Within this composition range, the orientation and masses of the ellipsoidal CBM does not appear to change significantly. For the selenide-rich alloys, the SdH measurements found a slight decrease of ~15 meV in the energy offset at *x* = 2.4 between the CBM (Γ) and the proposed second conduction band (*f*). The precise magnitude of the offset decrease could not be determined due to the error margins in the experiment. ARPES performed on an alloy crystal with a composition near Bi_2_Te_1.5_Se_1.5_ does not clearly show a conduction band pocket near *f*; however, there is a triangular Fermi surface centered at *k*_*x*_ = *k*_*y*_ = 0 which could be a trigonally warped *z* or Γ pocket [69].

Band structure calculations on the ordered Bi_2_Te_2_Se compound disagree regarding the order and location of the conduction band extrema. Reports can be found with the CBM occurring along the Γ*Z* line and a second edge along the *ZF* [70], the same relative locations but in reversed order [71–73], or having only a single valley at the Γ point [2]. These discrepancies arise due to the details of how these various calculations are performed and the sensitivity of results near the band gap to the effects of the spin-orbit interaction (SOI) [2, 17, 18]. Further, the true location of band extrema can be missed if calculations were only performed along high symmetry directions. This is problematic as detailed studies have found the CBM at *f* in Bi_2_Te_3_ to be near but not on the *ZF* line [1, 17, 74–80]. Evaluating the results of these calculations is complicated by a lack of experimental observations of the conduction band Fermi surface in the literature. ARPES has been performed on Bi_2_Te_2_Se; however, that study focused on the Dirac point in the surface states very near the VBM and did not capture the bulk conduction band [23].

In the absence of SOI, both the conduction band minimum (CBM) and valence band maximum (VBM) would be single valleys located at Γ, making these materials poor thermoelectrics and having no topological insulating properties. The CBM would consist primarily of Bi 6*p* states while the VBM would be comprised of Se 4*p* and Te 5*p* states. Instead, the SOI shifts the energy levels of these bands such that the Bi states and chalcogen states reverse their ordering at Γ [2, 19, 81, 82]. Where the bands overlap near Γ, an anticrossing occurs due to their opposite parity thereby opening a band gap. Now at Γ, the lowest energy conduction band states have chalcogen character and the valence band more Bi character. A schematic of the SOI-induced overlap effect is shown in Figure 3(b). In Bi_2_Te_3_, the overlap is significant enough to invert the curvature of the bands at Γ and shift the band extremum to a lower symmetry and higher valley degeneracy points. While the overlap does occur in Bi_2_Se_3_, it is not enough to invert the curvature at Γ and shift the extrema to different locations in the Brillouin zone [18, 19].

The magnitude of the SOI induced overlap increases monotonically from Bi_2_Se_3_ to Bi_2_Te_3_ when alloying [83]. Alloying between Bi_2_Te_3_ and Bi_2_Se_3_ can be qualitatively understood as modulating the magnitude of the SOI-induced band overlap. This is illustrated by electronic structure calculations in Figures 3(c)–3(f) for Bi_2_Se_3_ with varying degrees of SOI. Doubling the SOI for Bi_2_Se_3_ inverts the curvature at Γ creating the doubly degenerate valley at *z*. Furthermore, the energy level of the *z* pocket has moved above that of the sixfold degenerate pocket at *f*. Note that this band structure closely resembles that of Bi_2_Te_3_ where the SOI effect is greater (Figures 4(c) and 4(d)).

Knowing the effects of varying SOI allows for understanding of trends in the band gap with alloy composition. The opening of the band gap when substituting Te into Bi_2_Se_3_ results from the CBM at *Γ* moving higher in energy and the VBM lower due to the increased effects of the SOI-induced anticrossing. This explains why the band gap increases instead of the decrease expected when substituting less electronegative anions [84]. The peak in band gap near Bi_2_Te_2_Se is not caused by the crossing of the CBM of Bi_2_Te_3_ and the CBM of Bi_2_Se_3_ as has been previously suggested [85]. The *z* pocket is formed from the curvature inversion of Γ, and the crossing of *f* and Γ/*z* was found at *x* = 0.3 by SdH [68], coinciding with the observed peak in Seebeck effective mass (Figure 2). Any movement of the CB edge along Γ*a* also cannot explain the peak in band gap as it is calculated to be higher in energy than *f* and Γ/*z* for all compositions (Figure 3). Thus, the relative shifts of valence band edges must also be considered. In Bi_2_Te_3_, the VBM is located near the Γ*a* line with a second edge at *f*, while in Bi_2_Se_3_, the VBM is found at Γ. Note that the valence band at Γ also inverts and shifts its extremum to *z* in more Te-rich alloys. At some composition, the valley near the Γ*a* line and *f* valence band edge must cross the Γ/*z* and this may be the origin of the band gap peak. Recent interband absorption studies on this alloy system have found that at Bi_2_Te_2_Se, there is a change in the location of the lowest energy direct transition [55]. This may be a switch from the VB *f* to CB *f* transition in *x* < 1 to the VB Γ/*z* to CB Γ/*z* for *x* > 1. As the *f* and Γ/*z* cross at *x* = 0.3 in the conduction band, this would support a crossing between the valence band counterparts at *x* = 1.

## 5. Effective Mass Modeling of Conduction Band Electronic Transport

**Figure 5:**
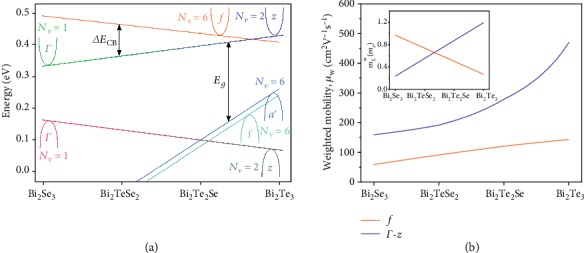
(a) Four band edges are primarily responsible for the electronic transport in the *n*-type Bi_2_Te_3−*x*_Se_*x*_ alloy system. The peak in the conduction band Seebeck effective mass near Bi_2_Te_2.7_Se_0.3_ occurs due to the crossing of the sixfold *f* and twofold *z* valleys. The loss of weighted mobility with increasing selenium content is attributable to the reduction in effective valley degeneracy due to the *f* pocket rising far above the CBM and increasing in mass and the *z* pocket transitioning from *N*_*v*_ = 2 to 1 as the curvature inverts at Γ due to the reduction in SOI-induced anticrossing. The peak in band gap near Bi_2_Te_2_Se can be understood as a crossing between the *a*′ and Γ/*z* valence band edges. This schematic can be used to fit a two-band effective mass model with weighted mobility and effective mass parameters as shown in (b). These parameters generate the dashed lines fit to transport data in Figure 2.

The shifts in band edge energies discussed in the previous section have been summarized schematically in Figure 5(a). With this picture established, an effective mass model can be fit that parameterizes the electronic transport in the *f* and Γ/*z* conduction band edges across the whole range of compositions. While charge transport in most materials is far more complex than that of nearly free electrons, an effective mass model that parameterizes the transport with empirical parameters equivalent to those of bands with a parabolic *E*(*k*) dispersion can make accurate predictions and provide useful physical insight [86]. The parameters of this model are determined empirically and should be interpreted as such.

The Seebeck coefficient, *S*; electrical conductivity, *σ*; weighted mobility, *μ*_*w*_; and Hall coefficient, *R*_*H*_, for carriers in a single band where acoustic phonon scattering dominates can be described using

(1) $$\begin{array}{c} S=\frac{k_{B}}{e}\left(\frac{2F_{1}}{F_{0}}-\eta \right), \end{array}$$

(2) $$\begin{array}{c} \sigma =\frac{8\pi e}{3}{\left(\frac{2m_{e}k_{B}T}{h^{2}}\right)}^{3/2}\mu_{w}F_{0}, \end{array}$$

(3) $$\begin{array}{c} \mu_{w}=\frac{\pi e\hbar^{4}C_{l}}{\sqrt{2}{\left(k_{B}T\right)}^{3/2}}\frac{N_{v}}{\Xi^{2}m_{c}^{*}}, \end{array}$$

(4) $$\begin{array}{c} R_{H}=\frac{3}{8\pi e}{\left(\frac{h^{2}}{2m_{S}^{*}k_{B}T}\right)}^{3/2}\frac{F_{-1/2}}{2F_{0}^{2}}. \end{array}$$

Here, *F*_*j*_ is a Fermi integral of order *j* and a function of the reduced chemical potential *η*, *C*_*l*_ is the average longitudinal elastic constant, *N*_*v*_ is the valley degeneracy, *Ξ* is the acoustic deformation potential, *m*_*c*_^∗^ is the conductivity effective mass, and *m*_*S*_^∗^ is the Seebeck effective mass. When multiple band edges are contributing to transport, their combined contributions can be expressed using

(5) $$\begin{array}{c} S=\frac{\sum_{i}S_{i}\sigma_{i}}{\sum_{i}\sigma_{i}}, \end{array}$$

(6) $$\begin{array}{c} \sigma =\underset{i}{\sum }\sigma_{i}, \end{array}$$

(7) $$\begin{array}{c} R_{H}=\frac{\sum_{i}R_{H,i}\sigma_{i}^{2}}{{\left(\sum_{i}\sigma_{i}\right)}^{2}}. \end{array}$$

A more complete effective mass model for this system would contain the variation with composition for each band of the Seebeck and conductivity masses, the deformation potentials, and the band degeneracies in addition to changes in lattice stiffness and alloy scattering energies. Unfortunately, most of these parameters have not been reported in the literature, and the large number of unknown variables leads to fit solutions which are not unique. Despite this difficulty, some conclusions may still be drawn regarding the band parameters.

Kohler reported that the lowest conduction band in Bi_2_Te_3_ was sixfold degenerate with a density-of-states mass of 0.27 *m*_e_ and a second conduction band was present only 25 meV higher having a much higher mass estimated to be ~3 *m*_e_ [64]. Single band edge effective mass modeling of an extensive amount of literature data for *n*-type Bi_2_Te_3_ found the relationship between conductivity and Seebeck could be well described by a weighted mobility of *μ*_*w*_ = 525 cm^2^V^−1^ s^−1^ and the Hall and Seebeck by a Seebeck effective mass of *m*_*S*_^∗^ = 1.06 *m*e [1]. If instead a two-conduction band model is fit to the same experimental data while using the masses and energy offsets reported by Kohler, the CBM at *f* has a *μ*_*w*_ = 182 cm^2^V^−1^ s^−1^ while the second minimum at *z* has *μ*_*w*_ = 437 cm^2^V^−1^ s^−1^. The exact ratio of the calculated weighted mobilities will depend upon the true mass and offset of the *z* pocket; however, a two-band system with a CBM of 0.27 *m*_e_ behaving like a single band with a mass of 1.06 *m*_e_ necessitates the second band having both a higher mass and weighted mobility. This result is contrary to expectation considering the *f* pocket has higher degeneracy and lower effective mass and *μ*_*w*_∝*N*_*v*_*Ξ*^−2^*m*_*c*_^∗−1^. This suggests that the acoustic deformation potential of the *f* pocket must be significantly larger than that of the *z*. When fitting a similar model for *n*-type Bi_2_Te_2.7_Se_0.3_, Konstantinov et al. found that the deformation potential of the sixfold pocket must be 6-8 times larger than the second, higher mass band edge [87]. Furthermore, the higher mass and weighted mobility implies that the large majority of carriers and nearly half of the electrical conductivity in *n-*type Bi_2_Te_3_ is attributable to the *z* pocket. This is a key departure from the typical description of the conduction band as simply being sixfold degenerate.

In Bi_2_Se_3_, the lowest band is singly degenerate at Γ with a band edge mass of 0.155 *m*_e_; however, the nonparabolicity of the band makes this a poor descriptor of the density-of-states beyond ~2∗10^18^ cm^−3^ carriers [67]. Instead, a slightly higher Seebeck mass would be more accurate for modeling Γ at thermoelectric doping levels. Single band effective mass modeling finds that a mass of 0.25 *m*_e_ accurately predicts the Seebeck data for Hall carrier concentrations between 5∗10^18^ and 1∗10^20^ cm^−3^ carriers. A potential second band edge was detected 160 meV above the CBM at a carrier concentration near 3∗10^19^ cm^−3^ carriers whose mass was only reported as “high” with no estimated value. The absence of any significant deviation with increased doping from the trend using the single band effective mass indicates that the CBM at Γ dominates transport in *n*-type Bi_2_Se_3_. The weighted mobility of any second band must be significantly lower than that of the Γ for a single band mass model to fit. Our electronic structure calculations predict this second band to be located at *f* (Figure 3(e)). As *f* is a sixfold degenerate point, the lower weighted mobility could result from some combination of a large conductivity mass or a large deformation potential.

We present in Figure 5(b) the result of a parametrization of the transport in this alloy system using a two-band effective mass model which follows the general trends in deformation potentials and masses previously discussed. The fitting parameters used for each band edge are the Seebeck effective mass, *m*_*S*_^∗^; the deformation potential, *Ξ*; and the ratio of the valley degeneracy to conductivity effective mass, *N*_*v*_*m*_*c*_^∗−1^. The valley degeneracy to conductivity mass ratio is not broken into its constituents as the value of valley degeneracy is not clearly defined for the Γ/*z* as it transitions between *N*_*v*_ = 1 in Bi_2_Se_3_ and *N*_*v*_ = 2 in Bi_2_Te_3_. Similarly, predicting the conductivity mass variations requires more significant knowledge than is available of pocket anisotropy changes with alloy composition. The values presented here are meant to serve as reasonable estimates based upon available data to be used for further experimental and theoretical verification.

Linear variations are assumed for fitting parameters between the binary compositions. Wherever possible, data from experimental results and band structure calculations are used to set parameters of the model. It must be noted that many of these experimental values were measured at temperatures lower than 300 K where the transport data was taken. Band offsets and masses could shift with temperature, and the degree to which this occurs is not clear. The values solved for in fitting are given in Table 1. These values were obtained by minimizing a sum of squared errors between the model and data points of Figure 2. The band gap and electronic transport in *p*- and *n*-type bismuth telluride alloys show important differences in compositional trends due to the details of their complex band dynamics. In Bi_2−*y*_Sb_*y*_Te_3_, a change in slope of band gap with composition coincides with a peak in Seebeck mass and weighted mobility when modeled using a single band [6, 13–15]. This correlation is explained by a crossing of two valence bands in energy with composition [6]. In Bi_2_Te_3−*x*_Se_*x*_, a peak in band gap does not coincide in composition with the peak in effective mass and no peak in weighted mobility is observed [4, 8, 16, 53, 54]. This suggests that the underlying causes differ for the mass and band gap peaks. The solid lines in this figure are simple guides to the eye, while the dashed lines represent single band evaluation of the simplified two-conduction band effective mass model discussed within the text and evaluated for a constant doping level of 5∗10^19^ cm^−3^.

We attempted to include in our model the effects of alloy scattering of charge carriers as described by Harrison and Hauser [11]; however, best fit values obtained were very small (<1 meV) and did not improve the model fit. We therefore exclude these effects from the model. It is not clear why alloy scattering does not play a prominent role in transport as it does in other semiconductor alloys [88]. One possibility is that deformation potential scattering by phonons is strong enough to minimize the impact of other scattering mechanisms. This appears to be the case for the lack of significant ionized impurity scattering near room temperature in Bi_2_Te_3_ due to the relatively soft lattice and large static dielectric permittivity [1]. The relaxation times for carrier scattering have *T*^−1/2^ and *T*^−3/2^ dependence for alloy and deformation potential scatter, respectively [11, 89]. The impact of alloy scattering therefore may be more important at temperatures below 300 K where the data used in this study was collected.

Shown in Figure 5(b) are the Seebeck masses and resulting weighted mobilities for the two conduction bands which produce the fits of Figure 2 (dashed curves). The weighted mobility of both pockets decreases with increasing Se content of the alloy, however, for different reasons. The *f* pocket presumably remains sixfold degenerate, but its mass increases (decreasing *N*_*v*_*m*_*c*_^∗−1^) leading to a reduction in mobility. The Γ/*z* pocket decreases in *N*_*v*_ from 2 in Bi_2_Te_3_ to 1 in Bi_2_Se_3_; however, its mass decreases substantially such that the weighted mobility would still be expected to increase. This suggests that the deformation potential of the Γ/*z* band increases from Bi_2_Te_3_ to Bi_2_Se_3_. Our model fits this increase as from 5 to 16 eV. An improved fit could be obtained by not assuming a linear variation in parameters across the entire alloy composition; however, experimental and theoretical data do not provide sufficient guidance to avoid overfitting. Future studies could refine the understanding of this important thermoelectric and topological insulator material system.

## 6. Thermal Conductivity of Bi_2_Te_3−*x*_Se_*x*_ Alloys

Much of thermoelectric material engineering pertains to reducing a material's lattice thermal conductivity while still maintaining a high weighted mobility. This is possible because of the difference in order of magnitudes of the mean free paths of charge carriers (small) and phonons (small to large). Nevertheless, most methods of reducing thermal conductivity also reduce carrier mobility somewhat, and evaluation of the success in balancing electrical and thermal engineering can be performed using the quality factor [3]. Successful reduction of lattice thermal conductivity involves introducing effective scattering across the entire range of phonon frequencies responsible for heat transport [90]. Alloying reduces thermal conductivity by enhancing phonon scattering due to the influence of mass fluctuations and localized strains. With a relaxation time proportional to *ω*^−4^, it is most effective at scattering high frequency phonons [7]. Note that the relaxation time of other phonon scattering mechanisms common in thermoelectrics has different frequency dependencies and thus scatters different wavelengths of phonons: boundary scattering *ω*^0^, dislocation core *ω*^−3^, and dislocation strain *ω*^−1^ [91]. These contributions are highly dependent upon material processing. As we are concerned with the pure material properties of these alloys, the data considered within this section is from single or highly oriented large grain polycrystals and thus should be influenced by alloy scattering alone.

**Figure 6:**
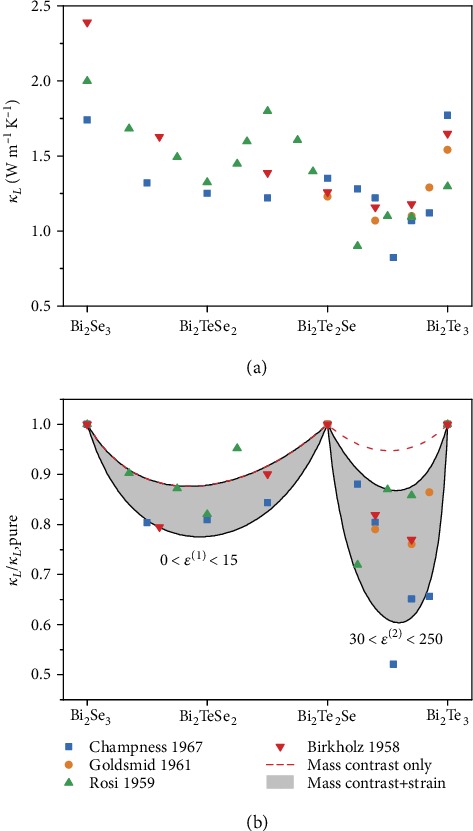
Alloying Bi_2_Te_3_ and Bi_2_Se_3_ significantly reduces the lattice thermal conductivity in comparison to the pure binaries and the ordered Bi_2_Te_2_Se phase [8, 9, 12, 16]. The absolute values of thermal conductivity (a) vary substantially among studies so the data is normalized to *κ*_*L*,pure_, Vegard's law interpolation between the lattice thermal conductivity of Bi_2_Te_3_ and Bi_2_Te_2_Se and between Bi_2_Te_2_Se and Bi_2_Se_3_ (b). Mass contrast alone nearly describes the trend between Bi_2_Te_2_Se and Bi_2_Se_3_; however, the lattice thermal conductivity Bi_2_Te_3_ and Bi_2_Te_2_Se requires considerations of strain or bonding changes at least an order of magnitude higher.

The effect on lattice thermal conductivity of alloying Bi_2_Te_3_ and Bi_2_Se_3_ is shown in Figure 6(a). There are large variations in the lattice thermal conductivity reported in the literature; however, most studies report a global minimum near Bi_2_Te_2.5_Se_0.5_. To gain further insight, the alloy scattering model of Callaway and von Baeyer and Klemens was fit to the data of each study as shown in Figure 6(b) [92, 93]. Such models have been useful in explaining the thermal conductivity trends in lead chalcogenides [3], bismuth antimony telluride [12], SiGe [94], and half Heuslers [95].

(8) $$\begin{array}{c} \frac{\kappa_{L}}{\kappa_{L}^{p}}=\frac{\tan^{-1}u}{u}, \end{array}$$

(9) $$\begin{array}{c} u^{2}=\frac{\pi^{2}\theta_{D}\Omega }{hv^{2}}\kappa_{L}^{p}\Gamma . \end{array}$$

The lattice thermal conductivity of the alloy (Equation (8)), *κ*_*L*_, is expressed as a function of the *κ*_*L*_^*p*^ of the “pure” compound which is the linear extrapolation between the two alloy endpoints, and of the disorder scattering parameter, *u*, which is defined in Equation (9). The disorder scattering parameter is a function of the Debye temperature, *θ*_*D*_; the average atomic volume, *Ω*; the average speed of sound, *v*; and the scattering parameter, Γ. This scattering parameter is found in multiple forms in the literature. Due to the occupancy preference of the chalcogen sites and the presence of an ordered compound at Bi_2_Te_2_Se, the Bi_2_Te_3-__*x*_Se_*x*_ alloy system can be viewed as two separate alloy systems: Bi_2_Te_3_-Bi_2_Te_2_Se and Bi_2_Te_2_Se and Bi_2_Se_3_. In order to account for alloying on two different chalcogen sublattices, we use the scattering parameter form of Yang et al. which was used to describe the effects of alloying on each site in ZrNiSn [95].

(10) $$\begin{array}{c} \Gamma =\Gamma_{M}+\Gamma_{S}, \end{array}$$

(11) $$\begin{array}{c} \Gamma_{M}=\frac{\sum_{i=1}^{n}c_{i}{\left(\bar{M_{i}}/\overset{̿}{M}\right)}^{2}f_{i}^{1}f_{i}^{2}{\left(\left(M_{i}^{1}-M_{i}^{2}\right)/\bar{M_{i}}\right)}^{2}}{\sum_{i=1}^{n}c_{i}}, \end{array}$$

(12) $$\begin{array}{c} \Gamma_{S}=\frac{\sum_{i=1}^{n}c_{i}{\left(\bar{M_{i}}/\overset{̿}{M}\right)}^{2}f_{i}^{1}f_{i}^{2}\varepsilon_{i}{\left(\left(r_{i}^{1}-r_{i}^{2}\right)/\bar{r_{i}}\right)}^{2}}{\sum_{i=1}^{n}c_{i}}. \end{array}$$

Here, the scattering parameter (Equation (10)) is broken into the summation of a mass fluctuation (Equation (11)), Γ_*M*_, and a strain field fluctuation parameter (Equation (12)), Γ_*S*_. The fluctuation parameters sum over all *n* sublattices, three for tetradymites, and the occupancy of the *i*-th sublattice having a degeneracy of *c*. Both are functions of the fractional occupancy, *f*_*i*_^*k*^, by species *k*; the average atomic mass of the sublattice, $\bar{M_{i}}$; and the average mass of the compound, $\overset{̿}{M}$. The mass fluctuation parameter varies with the difference in mass, *M*_*i*_, between the alloying species, and the strain field on the difference in atomic radius, *r*_*i*_, and a parameter *ε*. This *ε* parameter was introduced by Abeles who derived it being dependent upon the Grüneisen parameter, bulk modulus of the matrix and the sphere of the alloying atom, and Poisson's ratio. Exact calculation of all these parameters is difficult. Thus, in practice, *ε* is a phenomenological, adjustable parameter determined when fitting experimental data and ranges between 0 (no strain scattering) and 100 [3, 95]. The *ε* parameter arises from a slight modification of the original scattering parameter derivation of Klemens which instead contains a term related to the average stiffness constant of nearest neighbor bonds [7]. While it may appear arbitrary in usage, the magnitude of *ε* can be interpreted as a general indicator of the importance of strain scattering on lattice thermal conductivity due to alloying. This suggests strain engineering by careful alloying could be an avenue for further thermal conductivity reduction, as has recently been reported for Eu- and Mn-doped PbTe [96].

While there is discrepancy in the reported endpoint lattice thermal conductivities of Bi_2_Te_3_, Bi_2_Te_2_Se, and Bi_2_Se_3_, the least-squared-error fit *ε* parameters for each alloying range listed in Table 2 are similar. This is the case even for the data of Rosi et al. that found a local maximum in *κ*_*L*_ near Bi_2_Te_1.5_Se_1.5_ [9]. No other report to our knowledge has observed a similar maximum. The majority of Rosi et al.'s data still fall within the same bounds of the model as other studies which suggests the local maximum is an outlier. Using the average endpoint and fitting parameters for each study, a model was generated considering mass fluctuation and strain effects (Figure 6(b)). The key role of Se/Te site preference emerges from this analysis. Between 1 < *x* < 3 where the composition changes only the occupancy of the (1) site, the variation in lattice thermal conductivity is primarily due to mass fluctuation effects (red dashed curve). This is not the case for 0 < *x* < 1, where some effect of strain and/or changes in bonding must be present (shaded region indicating reasonable bounds). The inability of mass fluctuation scattering alone to explain alloying effects contrasts with the Bi_2_Te_3_-Sb_2_Te_3_*p*-type alloy where only mass fluctuation scattering is required [12, 97]. In the analogous compound Bi_2_Te_2_S, the more electronegative sulfur atom preferentially occupies the (2) site. At this composition, large bond angle strains prevent the formation of the ordered compound, and instead, some of the (2) site are occupied by Te [29]. Similar but smaller strains must occur when alloying Se on the (2) site as between Bi_2_Te_3_ and Bi_2_Te_2_Se.

## 7. Conclusion

Transport modeling of the *n*-type Bi_2_Te_3−*x*_Se_*x*_ alloy system proves more challenging than the *p*-type Bi_2−*y*_Sb_*y*_Te_3_ alloys and complicates engineering thermoelectric materials. Differences in the SOI between the telluride and selenide lead to changes in location of the conduction band minimum with composition as well as changes in valley degeneracy and effective mass of some band edges. We have summarized the current state of experimental and theoretical knowledge of these band dynamics and parametrized the transport using a simple effective mass model to supply estimates of their values. Reports on the lattice thermal conductivity in the *n*-type alloy have yielded unclear results regarding trends and location of minimum values. Using an alloy scattering model incorporating the ordered Bi_2_Te_2_Se compound provides clarity and identifies bond strain as a key factor between Bi_2_Te_3_ and Bi_2_Te_2_Se. It is our hope that the analyses provided here will aid future efforts to characterize and engineer *n-*type Bi_2_Te_3−*x*_Se_*x*_ alloys.

## 8. Experimental Methods

### 8.1. Electronic Structure Calculations

The Bi_2_Te_3_ (*x* = 0), Bi_2_Se_3_, and Bi_2_Te_2_Se (*x* = 1) compounds were taken in their trigonal crystal structure (space group R-3m) with experimental cell parameters from the Materials Project as shown in Table 3 [98].

For the composition *x* = 0.5, a 2 × 2 × 2 supercell was built starting from the primitive cell of composition *x* = 1. The supercell parameters were taken as a linear interpolation between the supercell parameters for *x* = 1 and *x* = 0 compositions. Then, four Se atoms were replaced with four Te atoms. Finally, a smaller primitive cell with only 10 atoms was found.

The electronic band structures were calculated with Density Functional Theory (DFT), using the Vienna Ab initio Simulation Package (VASP) [99, 100], using the Perdew-Burke-Ernzerhof (PBE) [101] generalized gradient approximation (GGA) functional and adopting the projector augmented-wave (PAW) [102, 103] approach. Spin-orbit interaction (SOI) was included in all the calculations to obtain the complexity of the band structure. Also, the source code of VASP was edited in order to change the weight of SOI in the case of Bi_2_Se_3_. In order to get a band structure that fits better the experimental band structure, we set a SOC weight equal to 0.7, corresponding to 100% in Figure 3. Van der Waals interactions were not included.

The band structure on the bisectrix plane for the Bi_2_Te_3_ and Bi_2_Se_3_, shown in Figure 4, was calculated with a non-self-consistent field calculation on a grid of 10,404 *k*-points.

The band structure on the high symmetry path, shown in Figure 4, was calculated using 50 *k*-points in each segment. The information about the atomic character of the bands is shown using colors.

The band structure of the Bi_2_Te_2.5_Se_0.5_ compound was calculated using BandUP software [104, 105]. This software allows to unfold the bands of a supercell back to the standard high symmetry path of the primitive cell. It is worth mentioning that the unfolding procedure returns a weight of the projection of the eigenvalues to the specific path. Thus, the band structure in Figure 4(b) is plotted in gray scale representing the weight of the projection. The same technique was used to plot the bands on the bisectrix plane, but only the maximal weight of the projection was considered. This allowed a plot in two dimensions while using color to represent the band energy. Also, to limit the computational cost, a 25 × 25*k*-point mesh covering only the region occupied by the two pockets of interest was used.

All data analyses, such as band structure plotting and supercell generation, were carried out using the pymatgen python package [106].

### 8.2. Effective Mass Modeling

In a single band system with a parabolic energy dependence on *k*, the empirically determined Seebeck mass and weighted mobility are independent of the doping level. In a system where multiple bands are contributing to transport, the Seebeck mass and weighted mobility determined using a single band model will vary with doping level. This complicates comparison as our goal in this report is to explain the variation of a single band effective mass with alloy composition. To allow for comparison, the dashed curves in Figure 2 were produced at a constant total carrier concentration of 5∗10^19^ cm^−3^. Charge neutrality was used to determine the Fermi level in the multiband system at this doping level and subsequently calculate the transport using Equations (1) through (7). Once the total Seebeck, conductivity, and Hall values were calculated using the multiband model, single band mass and weighted mobility parameters could be then calculated. Note that while a constant dopant concentration was used to produce the dashed curves of Figure 2, the multiband model parameters were best fit considering the various measured Hall values from the cited works.

## Figures and Tables

**Table 1:** Fitting parameters used in a two-conduction band effective mass model. Linear variation was assumed between the binary compositions.

	Seebeck mass, *m*_*S*_^∗^ (*m*_e_)		Deformation potential, *Ξ* (eV)		*N*_*v*_ *m*_*c*_^∗−1^ ratio (*m*_e_^−1^)	
	*f*	Γ/*z*	*f*	Γ/*z*	*f*	Γ/*z*
Bi_2_Se_3_	0.97	0.25	62.4	62.4	44.3	8.2
Bi_2_Te_3_	0.27	1.19	5.0	16.4	124.7	2.7

**Table 2:** Summary of “endpoint” lattice thermal conductivity data from various authors and the *ε* parameter used to produce a best fit for each author's data [8, 9, 12, 107].

Reference	Lattice thermal conductivity (W/mK)			Best fit *ε* parameter for chalcogen sublattice	
	Bi_2_Te_3_	Bi_2_Te_2_Se	Bi_2_Se_3_	Te/Se^(1)^	Te/Se^(2)^
Birkholz [8]	1.65	1.26	2.39	7.7	75.2
Rosi et al. [9]	1.30	1.24	2.00	0	72.8
Goldsmid [12]	1.54	1.23			82.7
Champness et al. [16]	1.77	1.35	1.74	13.7	169.3
Average	1.56	1.27	2.04	7.1	100.0

**Table 3:** Experimental cell parameters used for electronic structure calculations [11].

Compound	*a* = *b* = *c* (Å)	*α* = *β* = *γ* (°)	Volume (Å^3^)
Bi_2_Te_3_	10.468	24.164	168.933
Bi_2_Se_3_	9.841	24.304	141.890
Sb_2_Te_3_	10.284	23.851	156.217
